# Right ventricular dysfunction improves prediction of atrial fibrillation in hypertrophic cardiomyopathy: a cardiac magnetic resonance study

**DOI:** 10.3389/fcvm.2025.1587398

**Published:** 2025-06-05

**Authors:** Shengxu Li, Xuanye Bi, Quanxu An, Yuhang Li, Chenyao Li, Deliang Shen

**Affiliations:** ^1^ Department of Plastic Surgery, The Fifth Clinical Medical College of Henan University of Chinese Medicine (Zhengzhou People’s Hospital), Zhengzhou, China; ^2^ Department of Cardiology, Henan Province Key Laboratory of Cardiac Injury and Repair, The First Affiliated Hospital of Zhengzhou University, Zhengzhou, China

**Keywords:** hypertrophic cardiomyopathy, right ventricular dysfunction, atrial fibrillation, cardiac magnetic resonance, prognosis

## Abstract

**Background:**

Atrial fibrillation (AF) is a critical arrhythmia in hypertrophic cardiomyopathy (HCM), yet the role of right ventricular (RV) dysfunction in AF risk stratification remains underexplored. We aimed to evaluate the association between RV remodeling and incident AF in HCM patients.

**Methods:**

This retrospective cohort study included 612 HCM patients who underwent cardiac magnetic resonance (CMR) at our institution (2016–2023). Incident AF was identified via electronic medical records or structured telephone interviews. RV function was assessed using CMR-derived parameters, including ejection fraction (RVEF), peak emptying rate (PER), and peak filling rate (PFR).

**Results:**

Among 612 patients (66.1% male), 72 (11.8%) had preexisting AF, and 29 (5.4%) developed new-onset AF over a median follow-up of 3.3 years. Patients with AF (preexisting or new-onset) exhibited older age and impaired RV function at baseline, including reduced RVEF, PER, and PFR (*P* < 0.05 for all). Multivariable Cox regression identified age, left atrial diameter (LAD), RVEF, and RV-PFR as independent predictors of new-onset AF. Adding RVEF and RV-PFR to a clinical model (age, NYHA class III/IV, LAD) significantly improved risk stratification (NRI: 0.80, *P* < 0.01; IDI: 0.07, *P* < 0.01).

**Conclusions:**

RV dysfunction is prevalent in HCM patients with AF and provides incremental prognostic value for predicting new-onset AF beyond traditional clinical markers. These findings underscore RV functional assessment as a critical tool in AF risk stratification for HCM patients.

## 1 Introduction

Hypertrophic cardiomyopathy (HCM), a genetic disorder characterized by unexplained myocardial hypertrophy, is associated with a substantially elevated risk of atrial fibrillation (AF), occurring in approximately 20% of patients—four times higher than the general population (1, 2). Traditional predictors such as left atrial (LA) enlargement, age, and New York Heart Association (NYHA) functional class form the basis of the HCM-AF risk score, yet its predictive accuracy remains limited (3).

The historical focus on left-sided cardiac remodeling in HCM has overshadowed the significance of RV involvement. Recent studies highlight that right ventricular (RV) dysfunction is not merely a secondary phenomenon but a critical contributor to adverse outcomes in HCM (4, 5). Mechanistically, RV dysfunction in HCM may arise from shared myopathic processes affecting both ventricles, ventricular interdependence due to septal hypertrophy, or secondary pulmonary hypertension (6). These pathways create a substrate for atrial stretch, neurohormonal activation, and arrhythmogenesis—factors central to AF development (7).

Notably, RV functional indices such as peak emptying rate (PER) and peak filling rate (PFR) offer unique insights into diastolic and systolic reserve, which may precede structural RV changes (8). In non-HCM cohorts, RV dysfunction independently predicts AF incidence, suggesting its role as a systemic marker of cardiopulmonary pathology (9). Despite these advances, the interplay between RV remodeling and AF in HCM remains poorly characterized, leaving a critical gap in risk stratification strategies.

This study investigates RV functional parameters derived from cardiac magnetic resonance (CMR) as novel biomarkers to redefine AF risk stratification in HCM, emphasizing the underappreciated role of the right ventricle.

## 2 Methods

We included patients with HCM who underwent CMR examinations in our hospital from 2016 to 2023. The diagnosis of HCM was based on either a genetic diagnosis of HCM and LV wall thickness of ≥13 mm, or non-familial HCM patients with a LV wall thickness ≥15 mm, but no other cause of hypertrophy identified. HCM patients with poor CMR image quality, a history of ablation for supraventricular arrhythmias, a history of septal myectomy and alcohol septal ablation and severe primary valvular heart disease were excluded.

This study was carried out in accordance with the Declaration of Helsinki.Written informed consent was obtained from every patient. The study protocol was approved by the ethics committee of the first affiliated hospital of Zhengzhou University.

### 2.1 CMR protocol

CMR imaging was performed using a Siemens Skyra (3.0 T) magnetic resonance imaging system equipped with body coils comprising 16 channels. During the imaging procedure, four MRI-compatible electrodes were affixed between the right first and second ribs and the left fifth and sixth ribs of the subjects. Electrocardiogram (ECG) signals were acquired to synchronize the scanning at a specific time point. The epicardial and endocardial boundaries of the left ventricle (LV) and right ventricle (RV) myocardium were meticulously delineated throughout the complete cardiac cycle on every cine short-axis image. This facilitated the assessment of LV and RV end-diastolic and end-systolic volumes (EDV and ESV), end-diastolic diameter (EDD), ejection fractions (EF), and myocardial mass. Myocardial mass was computed by multiplying the volume of the myocardium estimated at end-diastole by the specific gravity of the myocardium (1.05 g/ml). The indices for EDV, ESV and mass were indexed to the individual's body surface area.

**Figure 1:**
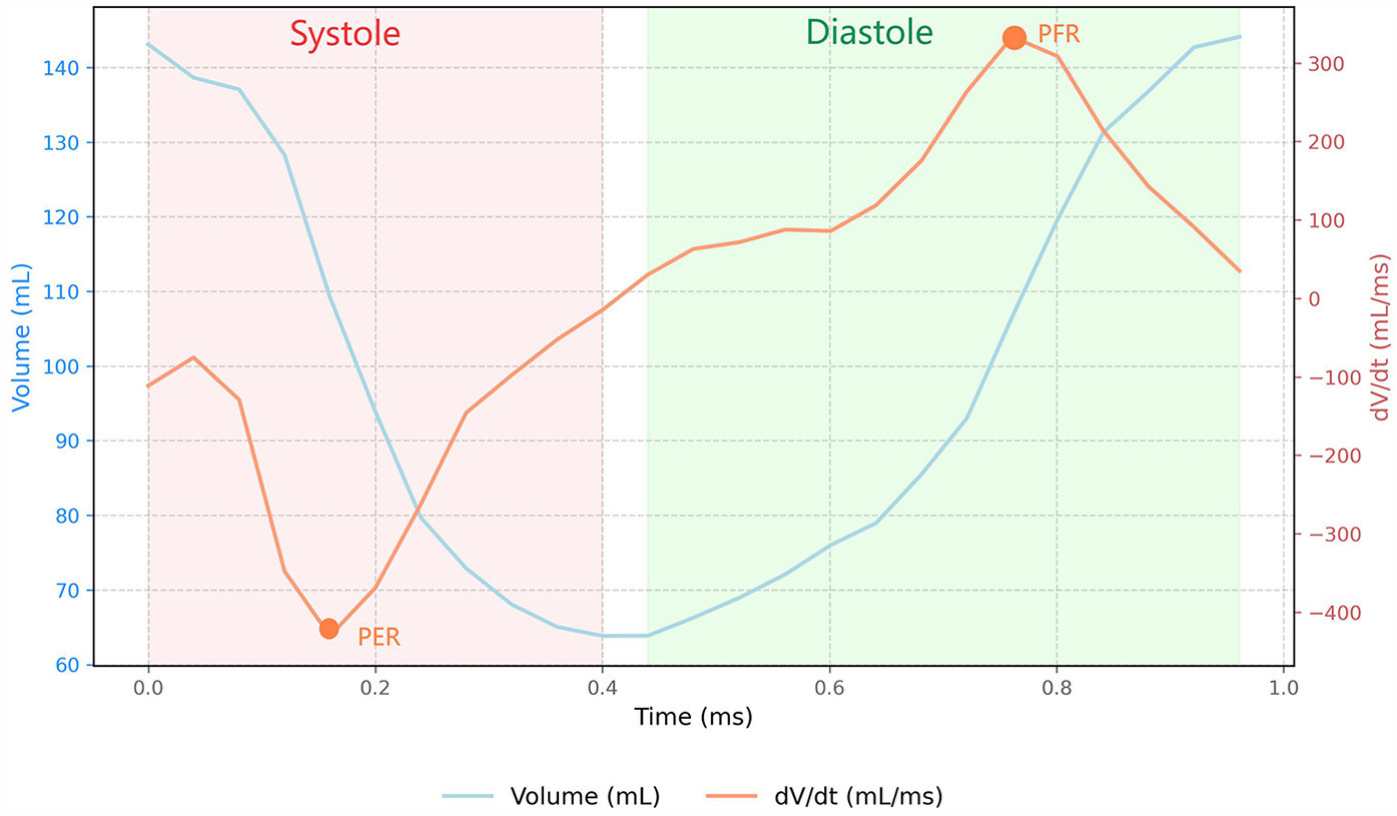
Right ventricular volume-time and dV/dt dynamics during cardiac cycle. The blue curve corresponds to the ventricular Volume-Time relationship, while the orange curve illustrates the corresponding dV/dt curve. The diastolic peak, identified as the peak filling rate (PFR), represents the maximum rate of ventricular filling during diastolic phase;The systolic peak, designated as the peak emptying rate (PER), indicates the maximum rate of ventricular volume reduction during systolic contraction.

Feature-tracking analysis was conducted using the CVI software (version 5.3.4, Circle Vascular Imaging), which offers precise bi-ventricular anatomical tracking capabilities. All post-processing analyses were performed by two independent cardiovascular imaging specialists with more than 5 years of dedicated CMR experience. Analysts were blinded to clinical outcomes and group assignments. The endocardial boundaries of left atrial (LA) was manually traced at the phase of the maximal LA volume before mitral valve opening and at the phase of the minimum LA volume after atrial contraction on 2-chamber and 4-chamber view. LAV max, LAV min and LAEF were derived from the CVI software. The indices for LAV max and LAV min were indexed to body surface area. RV global strain measurements were taken from the free wall. Global longitudinal strains (GLS) were derived from tracking the long horizontal axis cine images, while circumferential strains (GCS) and radial strains (GRS) were obtained from the short-axis cine images acquired through the standard CMR steady-state free precession sequence. The RV volume/time (V/t) and dV/dt curves were obtained plotting the cavity volumes over time (Figure 1). From the ventricular dV/dt curves, the peak during diastole was defined as the peak filling rate (PFR). PFR represents the maximum speed of RV filling. The peak during systole was defined as the peak empty rate (PER). PER represents the maximum speed of RV emptying. PFR and PER were normalized by the RV EDV, obtaining PFR index and PER index.

### 2.2 Patients follow-up

The new-onset AF during follow-up was identified through a review of hospital electronic medical records or a telephone interview with each study subject. AF was defined as an irregular heart rhythm without distinct *P*-waves documented on ECG, Holter registration (if duration ≥30 s), including any form of AF (paroxysmal, persistent, permanent).

### 2.3 Statistic analysis

Continuous variables are presented as mean ± SD and were analyzed by Student *t* test. When continuous variables had a skewed distribution, they were shown as median and interquartile range and were analyzed by Mann–Whitney *U* test. Categorical variables are presented as number (%) and were analyzed by the *χ*^2^ test. Receiver operating characteristic curves were used to determine the optimal cutoff for the new-onset AF based on the Youden index. Event rates are plotted in Kaplan–Meier curves for new-onset AF, and groups were compared using the log-rank test. Variables with *P* < 0.05 at univariate Cox proportional hazards models were included as covariables in multivariable models. Although NYHA III/IV was insignificant in univariate Cox analysis, we still included it in multivariable analysis as a well-recognized variable to predict AF in previous studies (3, 4). *Post-hoc* power analysis demonstrated 82% power to detect the observed RV-PFR difference (*α* = 0.05), supporting the robustness of our findings despite cohort size constraints.

To assess the incremental value of RV dysfunction in addition to conventional risk factors for predicting adverse events, we calculated the improvement in global *χ*^2^ values. Reclassification of patients was calculated using the category-free versions of the net reclassification index (NRI) and integrated discrimination improvement (IDI). Statistical analyses were performed using R software (Version 4.3.1; Package survivalROC and survIDINRI; R foundation for Statistical Computing, Vienna, Austria).

## 3 Results

The present study included 612 subjects, containing 357 (66.1%) males, 72 (11.8%) patients with preexisting AF and 29 (5.4%) patients with new-onset AF during a median follow-up of 3.3 years (2.3–3.8years). The baseline characteristics were summarized in Table 1.

**Table 1:** Demographic and clinical characteristics of HCM patients stratified by AF status.

Variables	Without preexisting AF	With preexisting AF	*P*. value	Without new-onset AF	With new-onset AF	*P*. value
	(*N* = 540)	(*N* = 72)		(*N* = 511)	(*N* = 29)	
Age (year)	53.0 (42.8, 62.0)	59.0 (53.8, 67.0)	**0** **.** **001**	53.0 (42.0, 61.0)	65.0 (55.0, 70.0)	**0**.**001**
Male (%)	357 (66.1%)	39 (54.2%)	0.063	340 (66.5%)	17 (58.6%)	0.500
NYHA III/IV (%)	95 (17.6%)	15 (20.8%)	0.611	88 (17.2%)	7 (24.1%)	0.483
Hypertention (%)	254 (47.0%)	38 (52.8%)	0.429	237 (46.4%)	17 (58.6%)	0.274
Diabetes (%)	67 (12.4%)	5 (6.9%)	0.247	60 (11.7%)	7 (24.1%)	0.093
Beta-blocker (%)	399 (73.9%)	50 (69.4%)	0.510	381 (74.6%)	18 (62.1%)	0.203
Calcium antagonist (%)	92 (17.0%)	9 (12.5%)	0.421	87 (17.0%)	5 (17.2%)	1.000

AF, atrial fibrillation; HCM, hypertrophic cardiomyopathy; NYHA, New York heart association class.

Bold values indicate statistically significant *P*-values.

### 3.1 RV function between HCM patients with and without AF

As showed in Table 2, in patients with preexisting AF, age and left atrial remodeling were significantly advanced compared to the non-AF group (all *P* < 0.05), with larger left atrial dimensions (LAD, LAVI max/min, all *P* < 0.01) and impaired LAEF (*P* = 0.001). Notably, this group exhibited pronounced RV dysfunction characterized by reduced systolic and diastolic performance (RVEF, *P* = 0.003; RV-PFR, *P* = 0.022), despite preserved RV size and strain parameters (*P* > 0.05).

**Table 2:** Comparative analysis of imaging parameters of HCM patients stratified by AF status.

Variables	Without preexisting AF	With preexisting AF	*P*. value	Without new-onset AF	With new-onset AF	*P*. value
	(*N* = 540)	(*N* = 72)		(*N* = 511)	(*N* = 29)	
Septal wall thickness (mm)	21.0 (18.0, 25.0)	20.0 (17.0, 24.3)	0.192	21.0 (18.0, 25.0)	20.0 (17.0, 25.0)	0.482
LAD (mm)	37.0 (32.0, 42.3)	45.0 (39.0, 53.0)	**0**.**001**	37.0 (32.0, 42.0)	41.0 (39.0, 47.0)	**0**.**002**
LAEF (%)	51.6 (44.0, 59.3)	36.5 (20.6, 54.2)	**0**.**001**	51.7 (44.6, 59.3)	48.3 (42.3, 60.7)	0.554
LAVImax (ml/m^2^)	51.1 (37.2, 89.5)	72.1 (50.2, 92.3)	**0**.**004**	50.7 (37.2, 85.2)	77.6 (39.0, 116)	0.141
LAVImin (ml/m^2^)	30.7 (19.5, 56.8)	53.2 (31.0, 69.1)	**0**.**001**	30.0 (19.4, 55.1)	44.0 (23.3, 76.1)	**0**.**023**
LVD (mm)	37.0 (32.0, 42.3)	45.0 (39.0, 53.0)	**0**.**001**	46.0 (42.0, 50.0)	43.0 (41.0, 49.0)	0.235
LVEF (%)	55.7 (43.0, 62.9)	52.9 (42.2, 61.3)	0.448	55.8 (43.0, 63.0)	50.0 (42.1, 58.5)	0.323
LVEDV index (ml/m^2^)	75.9 (63.6, 91.9)	73.9 (61.1, 91.5)	0.66	75.9 (63.7, 91.1)	77.0 (62.8, 112)	0.568
LVESV index (ml/m^2^)	32.2 (25.7, 42.1)	33.5 (25.0, 52.2)	0.192	32.2 (25.7, 41.4)	37.8 (27.7, 54.1)	0.284
LV mass index (g/m^2^)	69.3 (43.7, 94.9)	66.7 (50.8, 81.6)	0.547	69.6 (44.0, 96.3)	65.5 (32.7, 82.1)	0.244
RVD (mm)	31.0 (25.0, 36.0)	33.0 (27.8, 38.0)	0.079	31.0 (25.0, 36.0)	31.0 (26.0, 35.0)	0.699
RVEF (%)	41.8 ± 13.6	36.8 ± 13.4	**0**.**003**	42.3 ± 13.5	34.0 ± 13.0	**0**.**002**
RVEDV index (ml/m^2^)	60.3 (49.8, 72.4)	58.7 (50.7, 68.3)	0.389	61.0 (50.2, 72.9)	51.7 (42.0, 59.5)	**0**.**001**
RVESV index (ml/m^2^)	33.4 (26.6, 43.3)	35.6 (29.8, 42.8)	0.175	33.5 (26.9, 43.4)	31.9 (25.4, 40.6)	0.487
RV mass index (g/m^2^)	13.5 (11.2, 16.3)	14.2 (11.7, 15.5)	0.654	13.5 (11.2, 16.4)	11.9 (10.2, 15.1)	0.089
RV-PER (ml/s)	309 (219, 485)	245 (200, 348)	**0**.**001**	318 (225, 494)	220 (182, 274)	**0**.**001**
RV-PFR (ml/s)	225 (156, 399)	193 (148, 304)	**0**.**022**	230 (160, 410)	154 (90.6, 205)	**0**.**001**
RV-PER index (/s)	2.72 (2.10, 3.92)	2.34 (1.74, 3.04)	**0**.**001**	2.82 (2.12, 3.97)	2.28 (1.86, 3.01)	**0**.**024**
RV-PFR index (/s)	2.03 (1.46, 3.40)	1.73 (1.38, 2.61)	**0**.**018**	2.07 (1.48, 3.45)	1.54 (1.15, 2.16)	**0**.**002**
RV-RS (%)	24.0 (17.3, 31.5)	22.6 (17.8, 28.8)	0.492	24.1 (17.4, 31.7)	23.4 (16.9, 29.6)	0.448
RV-CS (%)	−13.9 (−17.5, −10.4)	−13.5 (−16.5, −11.1)	0.845	−14.0 (−17.5, −10.4)	−13.7 (−15.4, −10.3)	0.398
RV-LS (%)	−19.6 (−24.3, −14.1)	−18.6 (−22.2, −14.1)	0.326	−19.5 (−24.3, −14.0)	−20.6(−23.9, −15.8)	0.443

AF, atrial fibrillation; EDV, end-diastolic volume; ESV, end-systolic volume; EF, ejection fraction; GRS, global radial strains; GCS, global circumferential strains; GLS, global longitudinal strains; HCM, hypertrophic cardiomyopathy; LAD, left atrial diameter; LA, left artial; LAV, left artial volume; LAVI, left artial volume index; LV, left ventricular; RV, right ventricular; PER, peak emptying rate; PFR, peak filling rate.All volumetric parameters are indexed to body surface area.

Bold values indicate statistically significant *P*-values.

Among patients developing new-onset AF during follow-up, similar patterns emerged but with earlier-stage alterations: elevated baseline LAD (*P* = 0.002) and LAVI min (*P* = 0.023) coexisted with preserved LAEF (*P* = 0.18). RV functional decline was more globally apparent, involving both contractile and volumetric parameters (RVEF, *P* = 0.002; RVEDV, *P* = 0.001), alongside markedly depressed peak filling/emptying rates (RV-PFR, *P* = 0.001; RV-PER, *P* = 0.001).

### 3.2 Predictors of new-onset AF

In univariate analysis (Table 3), age, LAD, NYHA III/IV, RVEF, RVEDV index, RV-PER and RV-PFR predicted the new-onset AF in HCM patients. However, only age, LAD, RVEF, RV-PFR remained significant in multivariable analysis.

**Table 3:** Univariate and multivariable cox regression for new-onset AF in HCM.

Variables	Univariate analysis	*p* value	Multivariable analysis	*p* value
	HR (95% CI)		HR (95% CI)	
Age	1.05 (1.02–1.08)	0.001	1.05 (1.02–1.1)	0.006
NYHA III/IV	1.701 (0.73–3.99)	0.222	0.82 (0.33–2.03)	0.668
LAD	1.07 (1.03–1.12)	0.002	1.08 (1.03–1.13)	0.002
RVEF	0.96 (0.93–0.98)	0.001	0.96 (0.92–0.99)	0.011
RVEDV index	0.96 (0.93–0.98)	0.001	0.97 (0.94–1)	0.05
RV-PER	1 (0.99–1)	0.003	1 (1–1.01)	0.051
RV-PFR	0.99 (0.99–1)	0.001	0.99 (0.98–1)	0.014

AF, atrial fibrillation; CI, confidence interval; EDV, end-diastolic volume; EF, ejection fraction; HCM, hypertrophic cardiomyopathy; hazard ratio; LAD, left atrial diameter; NYHA, New York heart association class; RV, right ventricular; PER, peak emptying rate; PFR, peak filling rate.

**Figure 2:**
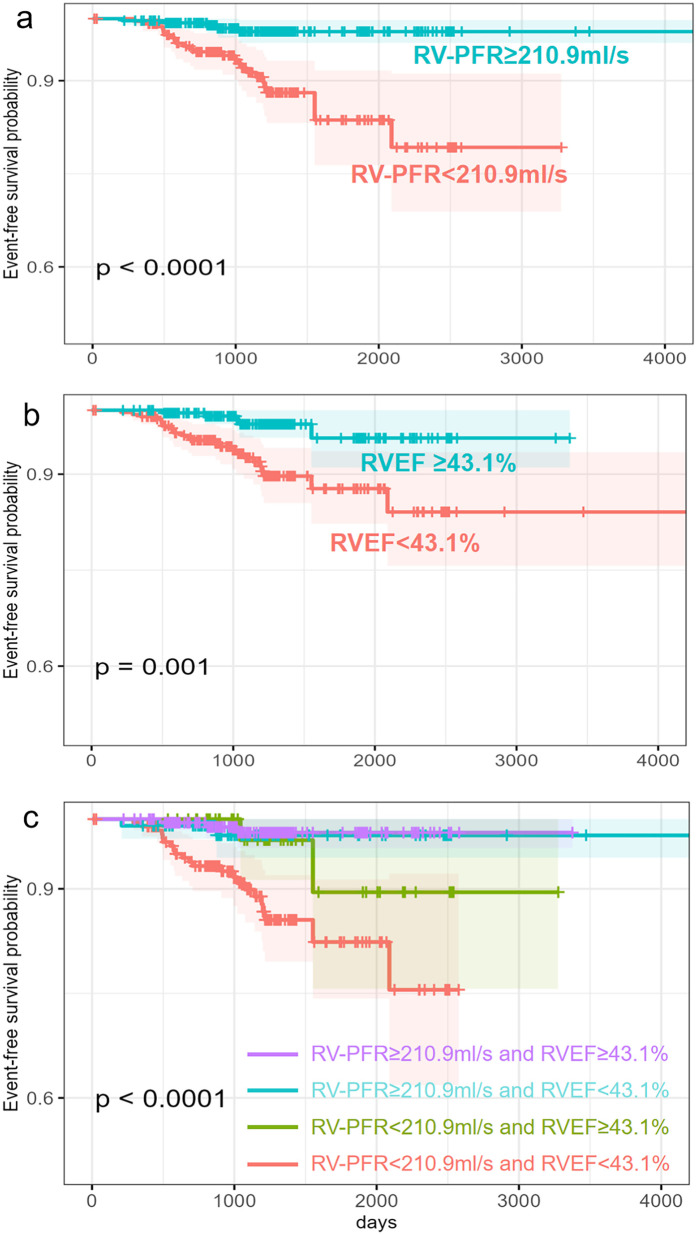
Kaplan–Merier survival analysis for new-onset AF in HCM patients. Patients are stratified by RVEF **(b)**, RV-PFR **(a)** and both RVEF and RV-PFR **(c)**.

The receiver operating characteristic analysis determined the optimal RVEF and RV-PFR cutoff was 43.1% and 210.9 ml/s respectively for predicting new-onset AF. Kaplan–Meier analyses of event-free survival for new-onset AF were shown in Figure 2. HCM patients with lower RVEF (<43.1%, Figure 2B) and lower RV-PFR (<210.9 ml/s, Figure 2A) were more likely to experience new-onset AF in the follow-up (both *p* < 0.05). In addition, HCM patients both with lower RVEF (<43.1%) and lower RV-PFR (<210.9 ml/s) showed the worst prognosis for new-onset AF (Figure 2C).

### 3.3 Incremental prognostic value of RVEF and RV-PFR for new-onset AF

**Figure 3:**
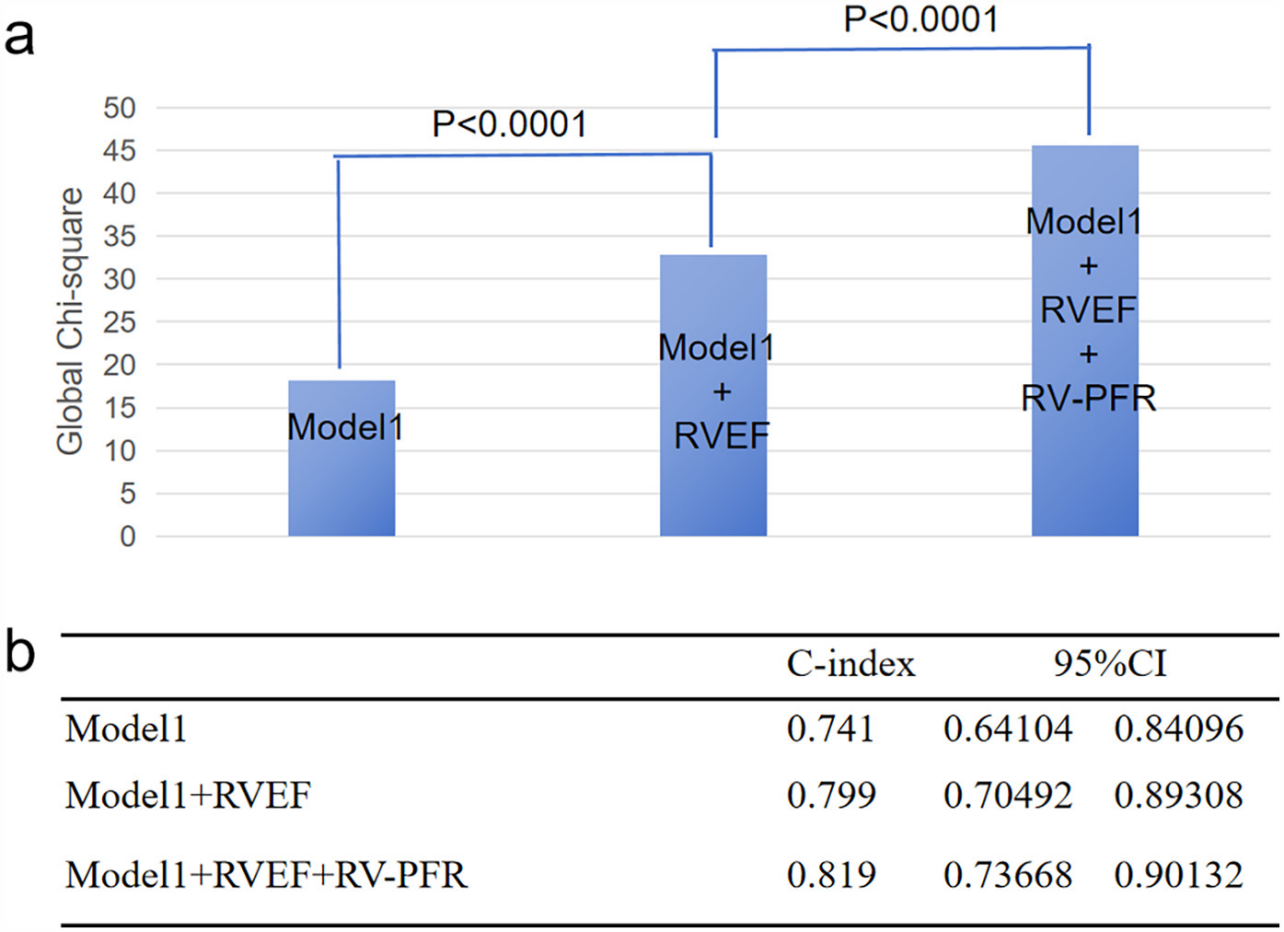
Incremental value of RVEF and RV-PFR for new-onset AF in HCM patients. The addition of RVEF and RV-PFR in sequence achieved incremental global *χ*^2^ **(a)** and C index **(b)** to known risk factors for the prediction of new-onset AF (age, NYHA class III/IV, LAD).

As showed in Figure 3, we added clinical variables, such as age, LAD, NYHA III/IV to basic model (global *χ*^2^, 18.21; C index 0.741). Addition of RVEF achieved an increase in the *χ*^2^ statistic (global *χ*^2^, 32.88; C index 0.799, *p* < 0.001; Table 4) and improved risk stratification [NRI, 0.6795 (0.3326–1.0264), *p* < 0.01; IDI, 0.0461 (0.0138–0.0784), *p* < 0.01]. Furthermore, adding RV-PFR achieved an additional increase in the *χ*^2^ statistic (global *χ*^2^ 45.54; C index 0.819, *p* < 0.001) and greater reclassification of patients [NRI, 0.8029 (0.4959–1.1099), *p* < 0.01; IDI, 0.0724 (0.0322–0.1126), *p* < 0.01; Table 4].

**Table 4:** Continuous NRI and IDI for new-onset AF by adding RVEF and RV-PFR to a model including clinical covariables.

Variable	Continuous NRI			IDI		
	NRI	95% CI	*p* value	IDI	95% CI	*p* value
Model + RVEF	0.6795	0.3326–1.0264	<0.001	0.0461	0.0138–0.0784	<0.001
Model + RVEF + RV-PFR	0.8029	0.4959–1.1099	<0.001	0.0724	0.0322–0.1126	<0.001

Baseline clinical model included age, New York Heart Association class III/IV, left atrial diameter.

AF, atrial fibrillation; CI, confidence interval; IDI, integrated discrimination improvement; NRI, net reclassification index; RVEF, right ventricular ejection fraction; PFR, peak filling rate.

## 4 Discussion

This study provides the a comprehensive analysis of the association between RV dysfunction and AF risk in patients with HCM. Our findings reveal three key insights: (1) RV functional impairment is prevalent in HCM patients with AF, (2) RVEF and RV-PFR independently predict new-onset AF, and (3) RV metrics significantly enhance AF risk stratification beyond conventional predictors. These discoveries fundamentally reshape our understanding of RV contributions to HCM pathophysiology.

### 4.1 RV functional assessment: a paradigm shift in HCM evaluation

Historically, RV function has been overshadowed by a predominant focus on LV pathology (10). Our findings challenge this LV-centric paradigm by demonstrating that 30% of HCM patients exhibit RV dysfunction—a prevalence mirroring recent CMR studies (11). Notably, Mahmod et al. (12) demonstrated that RV strain abnormalities precede LV systolic dysfunction in HCM, suggesting RV assessment could serve as an early disease marker. These observations position the RV not as a passive bystander, but as an active contributor to HCM progression. This RV involvement likely originates from three interconnected mechanisms: shared myopathic processes affecting both ventricles, ventricular interdependence due to septal hypertrophy, and secondary pulmonary hypertension (6, 13).

### 4.2 RV-PFR: overcoming limitations of conventional metrics

Current RV assessment tools present critical limitations. RVEF, while valuable for global systolic evaluation, proves load-dependent and insensitive to early dysfunction (14). Strain analysis suffers from modality-specific variability (15), and volumetric indices fail to capture functional decline. In this study RV-PFR quantifies the maximal early diastolic filling rate, reflecting ventricular compliance through direct measurement of pressure-driven atrial-ventricular gradients. Our results demonstrate that RV-PFR independently predicts new-onset AF (HR = 0.99, *P* = 0.014), with its addition to clinical models significantly improving reclassification accuracy (NRI = 0.80).This divergence may arise from their distinct pathophysiological correlates: RV-PFR directly capture pressure-mediated ventricular filling dynamics, whereas strain quantifies myocardial deformation magnitude. In HCM, early RV dysfunction manifests predominantly as impaired relaxation rather than contractile failure, favoring flow-derived metrics over deformation indices for AF prediction.

### 4.3 Mechanistic links between RV dysfunction and AF pathogenesis

The pathophysiological interplay between RV dysfunction and AF development in HCM involves a triad of interconnected mechanisms. Central to this relationship is hemodynamic coupling, where reduced RV peak filling rate directly elevates right atrial pressure through impaired ventricular compliance. This pressure overload induces atrial wall stretch, triggering electrical remodeling via calcium handling abnormalities and connexin dysregulation (16). Notably, this aligns with Chatterjee's observations in non-HCM populations, where RV dysfunction independently promoted AF initiation through similar pressure-mediated pathways (4). Neurohormonal activation further amplifies this process, as RV dysfunction correlates with elevated B-type natriuretic peptide (BNP) and pro-inflammatory cytokines such as IL-6 (17). These molecular mediators create a pro-fibrotic atrial microenvironment that disrupts intercellular conduction velocity and wavefront propagation. This mechanical strain propagates bidirectionally, exacerbating left atrial enlargement—the most robust traditional AF predictor (18, 19). Our multivariable analysis underscores this complex synergy, with RV-PFR (HR = 0.99) and left atrial diameter (HR = 1.08) operating through distinct but complementary pathways to drive AF progression.

### 4.4 Limitations and future directions

This study has limitations: (1) Retrospective, single-center design risks selection bias and underdetection of asymptomatic AF; (2) Small new-onset AF cohort (*n* = 29) limits multivariable model robustness; While the new-onset AF subgroup was modest (*n* = 29), *post-hoc* calculations indicated sufficient sensitivity (power = 82%) to identify RV dysfunction effects, reflecting the challenges of HCM cohort recruitment. (3) Lack of molecular data (e.g., genetic variants) to elucidate RV-AF pathways; (4) RV-PFR reproducibility across CMR platforms requires validation. Manual RV volumetric analysis remains time-intensive and lacks universal automation, limiting scalability. Future studies should employ prospective multicenter cohorts, integrate advanced imaging, and explore genotype-specific RV phenotypes.

## 5 Conclusions

RV dysfunction is prevalent in HCM patients with AF and provides incremental prognostic value for predicting new-onset AF beyond traditional clinical markers. These findings underscore RV functional assessment as a critical tool in AF risk stratification for HCM patients.

## Data Availability

The original contributions presented in the study are included in the article/Supplementary Material, further inquiries can be directed to the corresponding author.

## References

[1] Maron BJ Desai MY Nishimura RA Spirito P Rakowski H Towbin JA Diagnosis and evaluation of hypertrophic cardiomyopathy: JACC state-of-the-art review. J Am Coll Cardiol. (2022) 79(4):372–89. 10.1016/j.jacc.2021.12.002 35086660

[2] Alphonse P Virk S Collins J Campbell T Thomas SP Semsarian C Prognostic impact of atrial fibrillation in hypertrophic cardiomyopathy: a systematic review. Clin Res Cardiol. (2021) 110(4):544–54. 10.1007/s00392-020-01730-w 32880676

[3] Carrick RT Maron MS Adler A Wessler B Hoss S Chan RH Development and validation of a clinical predictive model for identifying hypertrophic cardiomyopathy patients at risk for atrial fibrillation: the HCM-AF score. Circ Arrhythm Electrophysiol. (2021) 14(6):e9796. 10.1161/CIRCEP.120.009796 34129346

[4] Chatterjee NA Shah RV Murthy VL Praestgaard A Shah SJ Ventetuolo CE Right ventricular structure and function are associated with incident atrial fibrillation: MESA-RV study (multi-ethnic study of atherosclerosis-right ventricle). Circ Arrhythm Electrophysiol. (2017) 10(1):e004738. 10.1161/CIRCEP.116.004738 28082528 PMC5261825

[5] Hiemstra YL Debonnaire P Bootsma M Schalij MJ Bax JJ Delgado V Prevalence and prognostic implications of right ventricular dysfunction in patients with hypertrophic cardiomyopathy. Am J Cardiol. (2019) 124(4):604–12. 10.1016/j.amjcard.2019.05.021 31204037

[6] Raman B Smillie RW Mahmod M Chan K Ariga R Nikolaidou C Incremental value of left atrial booster and reservoir strain in predicting atrial fibrillation in patients with hypertrophic cardiomyopathy: a cardiovascular magnetic resonance study. J Cardiovasc Magn Reson. (2021) 23(1):109. 10.1186/s12968-021-00793-6 34635131 PMC8504076

[7] Gersh BJ Maron BJ Bonow RO Dearani JA Fifer MA Link MS 2011 ACCF/AHA guideline for the diagnosis and treatment of hypertrophic cardiomyopathy: executive summary: a report of the American College of Cardiology foundation/American Heart Association task force on practice guidelines. J Am Coll Cardiol. (2011) 58(25):2703–38. 10.1016/j.jacc.2011.10.825 22075468

[8] Haddad F Doyle R Murphy DJ Hunt SA. Right ventricular function in cardiovascular disease, part II: pathophysiology, clinical importance, and management of right ventricular failure. Circulation. (2008) 117(13):1717–31. 10.1161/CIRCULATIONAHA.107.653584 18378625

[9] Severino S Caso P Cicala S Galderisi M de Simone L D'Andrea A Involvement of right ventricle in left ventricular hypertrophic cardiomyopathy: analysis by pulsed Doppler tissue imaging. Eur J Echocardiogr. (2000) 1(4):281–8. 10.1053/euje.2000.0043 11916607

[10] Mahmod M Raman B Chan K Sivalokanathan S Smillie RW Abd Samat AH Right ventricular function declines prior to left ventricular ejection fraction in hypertrophic cardiomyopathy. J Cardiovasc Magn Reson. (2022) 24(1):36. 10.1186/s12968-022-00868-y 35692049 PMC9190122

[11] Aziz EF Kukin M Javed F Musat D Nader A Pratap B Right ventricular dysfunction is a strong predictor of developing atrial fibrillation in acutely decompensated heart failure patients, ACAP-HF data analysis. J Card Fail. (2010) 16(10):827–34. 10.1016/j.cardfail.2010.05.004 20932465

[12] Lee S Park J Uhm J Kim JY Pak H-N Lee M-H Impact of atrial fibrillation on the clinical course of apical hypertrophic cardiomyopathy. Heart (British Cardiac Society). (2017) 103(19):1496–501. 10.1136/heartjnl-2016-310720 28428444

[13] Sørensen E Myrstad M Solberg MG Øie E Tveit A Aarønæs M. Right heart structure and function in lifelong recreational endurance athletes with and without paroxysmal atrial fibrillation. J Am Soc Echocardiogr. (2022) 35(12):1259–68. 10.1016/j.echo.2022.06.008 35760278

[14] Ventetuolo CE Lima JAC Barr RG Bristow MR Bagiella E Chahal H The renin-angiotensin system and right ventricular structure and function: the mesa-right ventricle study. Pulm Circ. (2012) 2(3):379–86. 10.4103/2045-8932.101657 23130107 PMC3487307

[15] Claus P Omar AMS Pedrizzetti G Sengupta PP Nagel E. Tissue tracking technology for assessing cardiac mechanics: principles, normal values, and clinical applications. JACC Cardiovasc Imaging. (2015) 8(12):1444–60. 10.1016/j.jcmg.2015.11.001 26699113

[16] Bi X Song Y Yang C Song Y Zhao S Qiao S Sex differences in atrial remodeling and its relationship with myocardial fibrosis in hypertrophic obstructive cardiomyopathy. Front Cardiovasc Med. (2022) 9:947975. 10.3389/fcvm.2022.947975 36531728 PMC9748677

[17] Ting P Chou A Wu VC-C Tsai F-C Chu J-J Chen C-Y Relationship between right ventricular function and atrial fibrillation after cardiac surgery. J Cardiothorac Vasc Anesth. (2017) 31(5):1663–71. 10.1053/j.jvca.2017.05.018 28826681

[18] Kawut SM Poor HD Parikh MA Hueper K Smith BM Bluemke DA Cor pulmonale parvus in chronic obstructive pulmonary disease and emphysema: the MESA COPD study. J Am Coll Cardiol. (2014) 64(19):2000–9. 10.1016/j.jacc.2014.07.991 25440095 PMC4347835

[19] Chahal H Heckbert SR Barr RG Bluemke DA Jain A Habibi M Ability of reduced lung function to predict development of atrial fibrillation in persons aged 45–84 years (from the multi-ethnic study of atherosclerosis-lung study). Am J Cardiol. (2015) 115(12):1700–4. 10.1016/j.amjcard.2015.03.018 25900353 PMC4450133

